# Incidence and risks for surgical site infection after closed tibial plateau fractures in adults treated by open reduction and internal fixation: a prospective study

**DOI:** 10.1186/s13018-020-01885-2

**Published:** 2020-08-24

**Authors:** Junyong Li, Yanbin Zhu, Kuo Zhao, Junzhe Zhang, Hongyu Meng, Zhucheng Jin, Jiangtao Ma, Yingze Zhang

**Affiliations:** 1 Department of Orthopaedic Surgery, The Third Hospital of Hebei Medical University, NO.139 Ziqiang Road, Shijiazhuang, Hebei 050051 People’s Republic of China; 2 Key Laboratory of Biomechanics of Hebei Province, Shijiazhuang, Hebei 050051 People’s Republic of China

**Keywords:** Closed tibial plateau fractures, Surgical site infection, Incidence, Risk factors

## Abstract

**Background:**

Surgical site infection (SSI) was the most common complication of tibial plateau fracture after open reduction and internal fixation (ORIF). Severe infections even required repeat surgeries, which would cause serious psychological harm to patients and increased the economic burden of treatment. In order to identify the characteristics of the SSI and to avoid the occurrence of SSI, we conducted a prospective study to investigate the incidence and independent risk factors of SSI after ORIF for closed tibial plateau fractures in adults.

**Methods:**

This study was performed at a first-level trauma center. From October 2014 to December 2018, the study subjects were adult patients with closed fractures of the tibial plateau, all of whom underwent open reduction and internal fixation (ORIF) treatment. Finally, a total of 1108 patients were followed up. We collected patient demographics information, surgery-related variables, and indexes from preoperative laboratory examinations. Univariate and multivariate logistic analysis models were used to investigate the potential risk factors.

**Results:**

Twenty-five patients (2.3%, 25/1108) developed SSI. A total of 15 of 25 infections (60.0%) were due to Staphylococcus aureus and 3 (12.0%) were due to MRSA. Independent risk factors of SSI identified by multivariate logistic analysis model were bone grafting: autograft (OR 6.38; 95% CI 2.155–18.886; *p* = 0.001) and allograft (OR 3.215; 95% CI 1.009–10.247; *p* = 0.048), fracture type (Schartzker V–VI) (OR 8.129; 95% CI 2.961–22.319; *p* < 0.001), aspartate aminotransferase (>40 U/L) (OR 5.500; 95% CI 2.191–13.807; *p* < 0.001), white blood cell (>10*10^9^/L) (OR 2.498; 95% CI 1.025–6.092; *p* = 0.044), and anion gap (>16 mmol/L) (OR 8.194; 95% CI 1.101–60.980).

**Conclusions:**

We should pay enough attention to patients who carried one or more of these factors at admission and adopt more reasonable treatment strategies to reduce or avoid the occurrence of SSI.

## Introduction

Tibial plateau fracture was one of the most common lower limb fractures in orthopedics, accounting for 36.5% of tibial and fibula fractures [1]. The tibial plateau was the weight-bearing area of the knee joint, if not treated properly, it would cause serious consequences. By far, ORIF is the most common treatment choice, which aims at anatomical reduction of fractures and restoration of the lower limb force line. However, there were many potential complications of surgical treatment after tibial plateau fracture, including SSI, joint stiffness, traumatic osteoarthritis, delayed, or non-union [2–4]. Among the complications, SSI was the most common one. Previous literature had reported that SSIs developed at 2–14.2% of patients after they underwent ORIF, half of which were deep infections [5–7]. Consequently, patients had to be re-admitted or even re-operated to replace or remove internal fixtures to treat the infection. Moreover, severe infections even required repeat surgeries, which would cause serious psychological harm to patients and increased the economic burden of treatment. Therefore, the prevention of postoperative infection was far more important than the treatment of postoperative infection itself. Accordingly, it was important to understand the characteristics and associated risk factors of SSI to reduce this complication.

Previous studies had been conducted to investigate the incidence and related risk factors of SSI, including the extended operation time, smoking status, open fracture type, higher level of fracture type (Schatzker types IV–VI), and so on [5–8]. However, most previous researches were designed as a retrospective, which might have limitations inaccuracy of data collection and obscure follow-up information.

Given the above, we designed this prospective study to describe the incidence and characteristics of SSI and to identify independent risk factors associated with SSI.

## Patients and methods

Data used in this study were extracted from the database of Surgical Site Infection in Orthopaedic Surgery (SSIOS), in which a prospective method was used to collect data on patients who underwent orthopedic surgeries between October 1, 2014 and December 31, 2018. In SSIOS study, surveillance of surgical site during hospitalization and telephone follow-up after discharge were conducted to identify surgical site infections.

In this study, the inclusion criteria were all adult patients (≥18 years) with closed tibial plateau fractures treated with ORIF. Multiple trauma patients were also included. Exclusion criteria were under 18 years of age, open fractures, pathological fractures caused by other diseases, treatment with external fixation or conservative method, incomplete medical data, patients lost to follow-up, and fractures around the prosthesis after knee replacement.

In order to accurately analyze the factors of postoperative infection, from the beginning, we collected as much information as possible about the patient, including patients’ demographics information, the preoperative evaluation, and the various indicators during the operation. We set up a well-trained team to collect the detailed information of each patient every day. Investigators visited the ward regularly, followed the patients closely, questioned them, looked at their medical records and charts, recorded the variables of interest, and examined the suture site for signs of infection since the day after the surgery. After discharge, all patients underwent regular telephone follow-up at postoperative 3, 6, and 12 months to determine the presence of SSI.

### Data collection of variables

Patients’ demographics information including age, gender, height, weight, chronic diseases (diabetes mellitus, hypertension, cerebrovascular disease, chronic heart disease), living places (rural or urban), history of any surgery, allergy to any medications, smoking status, alcohol consumption were extracted and documented.

Body mass index (BMI) was divided into four groups using Chinese standards: normal 18.5–23.9; underweight <18.5; overweight 24–27.9; obesity and morbid obesity ≥28.

Characteristics of fractures included injury mechanism (low or high-energy), side involved, combined injuries, and fracture classification (Schatzker classification system). Fall from a standing height was defined as a low-energy injury, and fall from a height, traffic accident, and a sports injury were defined as a high-energy injury.

Surgery-related variables included ASA grade (American Society of Anesthesiologists), preoperative duration, anesthesia pattern, operative duration, fixation type, intraoperative blood loss, intraoperative blood transfusion, bone grafting, intraoperative and postoperative intravenous use of antibiotic. Preoperative duration was defined as the time from injury to surgery and was divided into two groups: 1: ≤7 days and 2: >7 days. Anesthesia pattern was divided into regional anesthesia and general anesthesia. Operative duration was also divided into two groups: 1: ≤120 min and 2: >120 min. Intraoperative blood loss was divided into two groups: 1: ≤400 ml and 2: >400 ml. Bone grafting pattern was divided into autograft and allograft. Prophylactic antibiotics were administered intravenously 30 min before surgery according to guidelines [9].

We recorded the values of preoperative laboratory examinations and divided them into normal, higher, or lower than normal. These variables included platelet (PLT), albumin/globulin (A/G), alanine transaminase (ALT), neutrophils (NEUT), white blood cells (WBC), red blood cell (RBC), albumin (ALB), total cholesterol (TC), aspartate aminotransferase (AST), low-density lipoprotein (LDL-C), hematatocrit (HCT), lactate dehydrogenase (LDH), monocytes (MON), mean corpuscular hemoglobin (MCH), lymphocytes (LYM), hydroxybutyrate dehydrogenase (HBDH), triglyceride (TG), high density lipoprotein (HDL-C), very low-density lipoprotein (VLDL), γ-glutamyl transpeptidase (GGT), mean corpuscular volume (MCV), osmotic pressure (OSM), indirect bilirubin (IBIL), serum urea (UREA), uric acid (UA), hemoglobin (HGB), red cell distribution width (RDW), hypersensitive c-reactive protein (HCRP), platelet distribution width (PDW), glucose (GLU), mean corpuscular hemoglobin concentration (MCHC), total protein (TP), globulin (GLOB), anion gap (AG), aspartate total bilirubin (TBIL), direct bilirubin (DBIL), alkaline phosphatase (ALP), cholinesterase (CHE), and total bile acid (TBA).

### Definition of SSI

SSI was defined based on the standards of the Center for Disease Control (CDC) [10]. Fascia or muscle infections, skin dehiscence or persistent wound secretions, visible abscesses or gangrene requiring surgical debridement and implant exchange or removal were considered deep SSI. Infections limited to the skin of the surgical site, not exceeding the depth of the subcutaneous tissue, with common surgical incision problems (redness, swelling, pain) that could be cured by oral or intravenous antibiotics are considered superficial infections.

### Statistical analysis

For continuous variables, Student *t* test and Mann-Whitney *U* test were used (depending on whether the value of the variable is normally distributed), and the significance was *p* < 0.05. First, a univariate logic analysis was used to evaluate the relationship between each categorical variable and SSI. Then, the variables that were tested as significant in the univariate analyses to predict SSI were included in the multivariate logistic regression analysis model, and the independent predictors of SSI were finally determined. The goodness of fit of the model was tested using Hosmer-Lemeshow. *p* > 0.05 was acceptable goodness of fit.

## Results

### Characteristics of the study sample

**Fig. 1:**
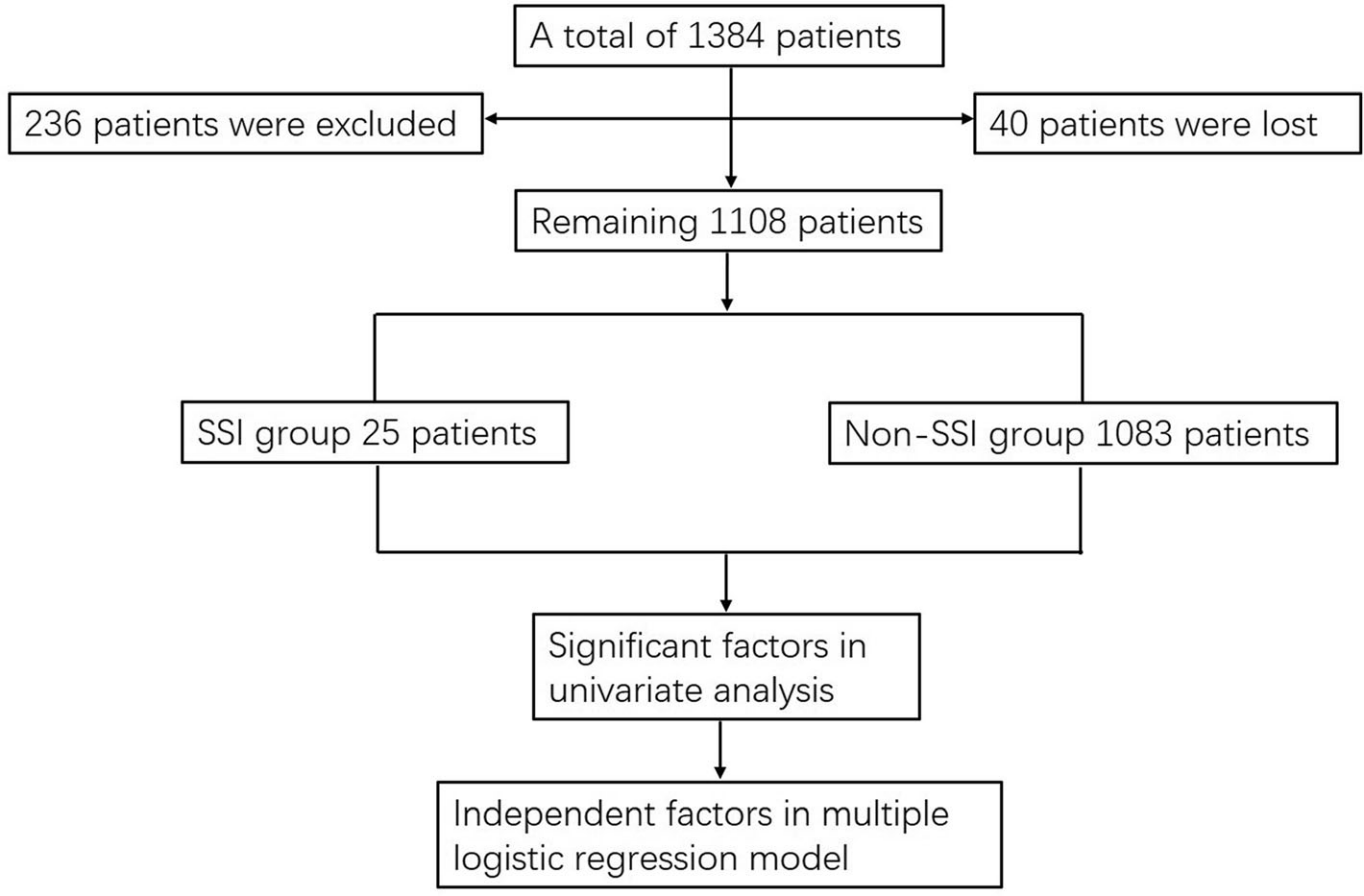
The flow chart for the selection of study participants

During the study interval, a total of 1384 patients with tibial plateau fracture were collected, 236 patients were excluded due to age less than 18 years (49), periprosthetic fracture (13), pathological fracture (14), open fracture (71), treatment with external fixation or conservative method (60), incomplete medical data (29), and patients lost to follow-up (40) (Fig. 1). Finally, 1108 patients were included in the final analysis, with a mean age of 45.6 years (range 18 to 82). There were 697 males and 411 females, with left side involved in 590 and right side in 518 cases.

### Characteristics of SSI

During the follow-up period, 25 (2.3 %) patients developed SSI after ORIF. Among them, 4 cases were cured by intravenous antibiotics and conventional local wound care, 12 cases were debrided in the operating room, and 9 cases were debrided combined with internal fixation hardware removal. The earliest diagnosis of SSI occurred on the 3 days post-operation, the latest on the 300 days, with a median time of 7 days. Each patient underwent wound secretion bacterial culture. *Staphylococcus aureus* was the most common causative pathogen (15, 60.0 %), of which 3 cases (3, 12.0%) were MASA, followed by *Escherichia coli* (3, 12.0%), *Enterobacter cloacae* (2, 8.0 %), *Acinetobacter baumannii* (1, 4.0%), *Pseudomonas aeruginosa* (1, 4.0%), multi-bacterium (1, 4.0%), and no pathogenic bacteria were cultured in 2 cases (2, 8.0%).

Statistically significant demographical and perioperative variables, such as age (45.6 versus 43.0 years, *p* = 0.347), preoperative stay (7.3 versus 14.5 days, *p* < 0.001), intraoperative blood loss (280.2 versus 434.0 ml, *p* = 0.005), operation duration (147.4 versus 195.8 min, *p* < 0.001), and hospital stay (19.3 versus 43.4 days, *p* < 0.001) were presented in Table 1. In terms of other variables, there was no significant difference between the SSI group and the non-SSI group. SSI prolonged a mean of 24.1 days of hospitalization than that of non-SSI.

**Table 1:** Comparison of continuous variables in patients with and without SSI

Variables	Patient without SSI (mean, standard deviation) (*n* = 1083)	Patient with SSI (mean, standard deviation) (*n* = 25)	*P*
Age (years)	45.6 (13.6)	43.0 (11.8)	0.347
Preoperative stay (days)	7.3 (6.9)	14.5 (17.9)	0.000*
Intraoperative blood loss (ml)	280.2 (267.4)	434.0 (370.7)	0.005*
Operation duration (minutes)	147.4 (64.7)	195.8 (68.4)	0.000*
Hospital stay (days)	19.3 (22.5)	43.4 (46.5)	0.000*

significant variables

### Univariate and multivariate analysis

Factors that significantly increased the risk of SSI in univariate analysis are summarized in Table 2, including patients’ demographics information, BMI, surgery-related variables, the values of preoperative laboratory examinations.

**Table 2:** Univariate analyses of risk factors associated with SSI after ORIF of closed tibial plateau fracture

Variables	Number (%) of SSI (*n* = 25)	Number (%) of non-SSI (*n* = 1083)	*P*
Gender (male)	20 (80.0)	677 (62.5)	0.074
Diabetes mellitus	2 (8.0)	150 (13.8)	0.401
Hypertension	2 (8.0)	197 (18.2)	0.189
Cerebrovascular disease	1 (4.0)	18 (1.7)	0.373
Chronic heart disease	1 (4.0)	51 (4.7)	0.868
History of any surgery	1 (4.0)	118 (10.9)	0.271
Allergy to any medications	3 (12.0)	138 (12.7)	0.912
Living area			0.827
Rural	15 (60.0)	673 (62.1)	
Urban	10 (40.0)	410 (37.9)	
Preoperative duration (days)			0.000*
1–7	8 (32.0)	723 (66.8)	
>7	17 (68.0)	360 (33.2)	
Anesthesia (general)	10 (40.0)	450 (41.6)	0.876
Number of fracture			0.057
1	11 (44.0)	679 (62.7)	
≥2	14 (56.0)	404 (37.3)	
Mechanism (high-energy)	17 (68.0)	676 (62.4)	0.569
Current smoking	4 (16.0)	147 (13.6)	0.727
Alcohol consumption	2 (8.0)	101 (9.3)	0.821
Bone grafting (yes)	13 (52.0)	225 (20.8)	0.000*
Bone graft type			0.000*
Autograft	8 (32.0)	113 (10.4)	
Allograft	5 (20.0)	112 (10.3)	
Operative duration (minutes)			0.001*
1–120	3 (12.0)	505 (46.6)	
>120	22 (88.0)	578 (53.4)	
Intraoperative blood loss (ml)			0.000*
1–400	15 (60.0)	931 (86.0)	
>400	10 (40.0)	152 (14.0)	
ASA class			0.942
I	3 (12.0)	155 (14.3)	
II	18 (72.0)	768 (70.9)	
III or above	4 (16.0)	160 (14.8)	
Prophylactic antibiotics use	22 (88.0)	1038 (95.8)	0.057
Postoperative antibiotics use	22 (88.0)	1005 (92.8)	0.362
Fixation type			0.774
Plate and screws	23 (92.0)	1012 (93.4)	
Screws only	2 (8.0)	71 (6.6)	
Intraoperative blood transfusion	6 (24.0)	106 (9.8)	0.020*
Fracture type (Schartzker)			0.000*
I–IV	6 (24.0)	835 (77.1)	
V–VI	19 (76.0)	248 (22.9)	
Age (years)			0.461
18–44	15 (60.0)	514 (47.5)	
45–64	8 (32.0)	462 (42.7)	
≥65	2 (8.0)	107 (9.9)	
BMI (kg/m^2^)			0.361
18.5–23.9	9 (36.0)	473 (43.7)	
<18.5	1 (4.0)	11 (1.0)	
24–27.9	8 (32.0)	383 (35.4)	
≥28.0	7 (28.0)	216 (19.9)	
TP (<60 g/L)	11 (44.0)	285 (26.3)	0.048*
ALB (<35 g/L)	12 (48.0)	224 (20.7)	0.001*
GLOB (<20 g/L)	4 (16.0)	157 (14.5)	0.833
A/G values			0.009*
1.2–2.4	18 (72.0)	970 (89.6)	
<1.2	6 (24.0)	81 (7.5)	
>2.4	1 (4.0)	32 (3.0)	
ALT (>40 U/L)	12 (48.0)	199 (18.4)	0.000*
AST (>40 U/L)	11 (44.0)	140 (12.9)	0.000*
TBIL (>21 μmol/L)	4 (16.0)	91 (8.4)	0.180
DBIL (>6 μmol/L)	9 (36)	302 (27.9)	0.372
IBIL (>14 μmol/L)	6 (24)	162 (15.0)	0.213
ALP (>100 U/L)	3 (12.0)	50 (4.6)	0.087
GGT (>60 U/L)	7 (28.0)	183 (16.9)	0.145
CHE (>12 kU/L)	1 (4.0)	27 (2.5)	0.635
TBA (>10 μmol/L)	2 (8.0)	65 (6.0)	0.679
HCRP (>8 mg/L)	17 (68.0)	732 (67.6)	0.965
LDH (>250 U/L)	10 (40.0)	244 (22.5)	0.040*
HBDH (>182 U/L)	5 (20.0)	183 (16.9)	0.683
TC (>5.2 mmol/L)	1 (4.0)	127 (11.7)	0.232
TG (>1.7 mmol/L)	3 (12.0)	186 (17.2)	0.496
HDL-C (<1.1 mmol/L)	12 (48.0)	435 (40.2)	0.430
LDL-C (>3.37 mmol/L)	2 (8.0)	148 (13.7)	0.413
VLDL (>0.78 mmol/L)	2 (8.0)	181 (16.7)	0.246
Na+ (<135 mmol/L)	13 (52.0)	320 (29.5)	0.015*
K+ (mmol/L)			0.772
3.5–5.5	24 (96.0)	1002 (92.5)	
<3.5	1 (4.0)	70 (6.5)	
>5.5	0 (0.0)	11 (1.0)	
CL- (mmol/L)			0.151
99–110	18 (72.0)	929 (85.8)	
<99	6 (24.0)	135 (12.5)	
>110	1 (4.0)	19 (1.8)	
TCO2 (mmol/L)			0.475
20–30	25 (100.0)	1022 (94.4)	
<20	0 (0.0)	17 (1.6)	
>30	0 (0.0)	44 (4.1)	
GLU (>6.1 mmol/L)	9 (36.0)	373 (34.4)	0.871
UREA (>8 mmol/L)	0 (0.0)	74 (6.8)	0.176
UA (>upper limit)	1 (4.0)	85 (7.8)	0.477
WBC (>10*10^9^/L)	15 (60.0)	344 (31.8)	0.003*
NEUT (1.8–6.3*10^9^/L)			0.281
1.8–6.3	9 (36.0)	556 (51.3)	
<1.8	0 (0.0)	6 (0.6)	
>6.3	16 (64.0)	521 (48.1)	
LYM (<1.1*10^9^/L)	5 (20.0)	303 (28.0)	0.379
MON (>0.6*10^9^/L)	15 (60.0)	658 (60.8)	0.939
RBC <lower limit	17 (68.0)	436 (40.3)	0.005*
HGB <lower limit	21 (84.0)	587 (54.2)	0.003*
HCT <lower limit	22 (88.0)	675 (62.3)	0.009*
MCV (fL)			0.365
82–100	25 (100.0)	1002 (92.5)	
<82	0 (0.0)	40 (3.7)	
>100	0 (0.0)	41 (3.8)	
MCH (pg)			0.564
27–34	24 (96.0)	990 (91.4)	
<27	0 (0.0)	47 (4.3)	
>34	1 (4.0)	46 (4.2)	
MCHC (g/L)			0.423
316-354	23 (92.0)	1008 (93.1)	
<316	0 (0.0)	32 (3.0)	
>354	2 (8.0)	43 (4.0)	
PDW (%)			0.557
12–18.1	21 (84.0)	945 (87.3)	
<12	4 (16.0)	116 (10.7)	
>18.1	0 (0.0)	22 (2.0)	
D-Dimer (>0.5 mg/L)	20 (80.0)	650 (60.0)	0.043*
AG (mmol/L)			0.024*
8–16	16 (64.0)	854 (78.9)	
<8	7 (28.0)	212 (19.6)	
>16	2 (8.0)	17 (1.6)	
OSM < 260 mOsm/L	6 (24.0)	104 (9.6)	0.017*
CA (mmol/L)			0.480
2.11–2.52	17 (68.0)	822 (75.9)	
<2.11	8 (32.0)	246 (22.7)	
>2.52	0 (0.0)	15 (1.4)	
P (mmol/L)			0.027*
0.85–1.51	17 (68.0)	932 (86.1)	
<0.85	2 (8.0)	53 (4.9)	
>1.51	6 (24.0)	98 (9.0)	
Mg (mmol/L)			0.008*
0.75–1.02	18 (72.0)	941 (86.9)	
<0.75	6 (24.0)	79 (7.3)	
>1.02	1 (4.0)	63 (5.8)	
PLT (10*10^9^/L)			0.905
125–350	20 (80.0)	901 (83.2)	
<125	1 (4.0)	41 (3.8)	
>350	4 (16.0)	141 (13.0)	

Abbreviation and notes: *ASA* American Society of Anesthesiologists; *BMI* body mass index; *RBC* red blood cell, reference range: female 3.5–5.0*10^12^/L; males 4.0–5.5*10^12^/L. *HGB* hemoglobin, reference range: females 110–150 g/L; males 120–160 g/L; *HCT* hematocrit, 40–50%; *MCV* mean corpuscular volume; *MCH* mean corpuscular hemoglobin; *MCHC* mean corpuscular hemoglobin concentration; *WBC* white blood cell; *NEUT* neutrophile; *LYM* lymphocyte; *MON* mononuclear cell; *PLT* platelet; 100–300*10^9^/L; *TP* total protein; *ALB* albumin; *GLOB* globulin; *A/G* values albumin/globulin; *ALT* alanine transaminase; *AST* aspartate aminotransferase; *TBIL* total bilirubin; *DBIL* direct bilirubin; *IBIL* indirect bilirubin; *ALP* alkaline phosphatase; *GGT* γ-glutamyl transpeptidase; *CHE* cholinesterase; *TBA* total bile acid; *HCRP* hypersensitive c-reactive protein; *LDH* lactate dehydrogenase; *HBDH* hydroxybutyrate dehydrogenase; *TC* total cholesterol; *TG* triglyceride; *HDL-C* high-density lipoprotein; *LDL-C* low-density lipoprotein; *VLDL* very-low-density lipoprotein; *GLU* glucose; *UREA* serum urea; *UA*, uric acid; *RDW* red cell distribution width; *PDW* platelet distribution width; *AG* anion gap; *OSM* osmotic pressure

*Significant variables

The multivariate analysis results showed that fracture type (Schartzker V–VI) (*p* < 0.001, OR = 8.129), bone grafting (*p* = 0.002), autograft (*p* = 0.001, OR = 6.380), allograft (*p* = 0.048, OR = 3.215), white blood cell (>10*10^9^/L) (*p* = 0.044, OR = 2.498), aspartate aminotransferase (>40 U/L) (*p* < 0.001, OR = 5.500), and anion gap (>16 mmol/L) (*p* = 0.040, OR = 8.194) were identified to be associated with SSI (Table 3).

**Table 3:** Multivariate analysis of factors associated with SSI after ORIF of closed tibial plateau fracture

Variables	OR	95%CI (lower limit)	95%CI (upper limit)	*P*
Preoperative duration	2.282	0.880	5.915	0.090
Bone grafting(yes)				0.002*
Autograft	6.380	2.155	18.886	0.001*
Allograft	3.215	1.009	10.247	0.048*
Operative duration (minutes)	3.544	0.958	13.108	0.058
Fracture type (Schartzker V–VI)	8.129	2.961	22.319	0.000*
AST (>40 U/L)	5.500	2.191	13.807	0.000*
WBC (>10*10^9^/L)	2.498	1.025	6.092	0.044*
AG (mmol/L)				0.078
<8	1.878	0.694	5.082	0.214
>16	8.194	1.101	60.980	0.040*

Abbreviation and notes: *AST* aspartate aminotransferase; *WBC* white blood cell; *AG* anion gap

*Significant variables

## Discussion

The incidence of SSI after ORIF varied according to different study designs and definitions of SSI. In this study, we designed it as prospective, with the purpose of better solving this problem and obtaining more reliable conclusions. This study showed that the overall incidence of SSI was 2.3% after ORIF of closed tibial plateau fractures in adults. Independent risk factors included bone grafting (autograft, allograft), fracture type (Schartzkern V–VI), aspartate aminotransferase (AST), white blood cell (WBC) count, and anion gap (AG).

We had come to a conclusion that fracture type (Schartzker V–VI) was an important predictor of SSI after ORIF of closed tibial plateau fractures in adults. The concept of fracture type (Schartzker V–VI) affecting the SSI rate after ORIF has been well studied in previous investigations [11–13]. Morris et al. investigated 302 patients undergoing ORIF of tibial plateau fractures and observed a deep infection rate of 22.3%, which suggested that Schartzker V–VI fractures have a significant independent effect on SSI [14]. Clinically, Schartzker V–VI fractures were usually found in high-energy injury including falls from a height, a traffic accident, and a sports injury [11]. Although a few previous studies had directly quantified this observed relationship, the level of fracture classification could reflect the severity of soft tissue injury to a certain extent [15, 16]. In addition, we theorize that fracture type (Schartzker V–VI) probably meant greater technical difficulty, prolonged operative time, and extended exposure of the wound, all of which might increase the chance of bacterial colonization and result in a higher incidence of SSI [17].

The tibial plateau fracture often was accompanied by compression of the fracture segments and collapse of the articular surface, which generally required bone grafting to restore the articular surface and maintain internal fixation until bone union. In the present study, autograft and allograft were used in 10.9% (121/1108) and 10.6% (117/1108) of the patients, and the incidence of SSI among them were 6.6% (8/121) and 4.3% (5/117), which were consistent with previously reported figures. In a retrospective of 198 patients undergoing surgery of tibial plateau fracture, Bagherifard et al. [18] found the complication rate of autograft and allograft was comparable, and they compared the SSI rate of autograft (4.3%, 1/23) and allograft (3.4%, 2/58). Lee et al .[19] analyzed the data from 1,303,347 patients who underwent primary bone grafting and the infection rate was 3.05%, demonstrating a higher SSI incidence rate in patients who received autologous bone grafts (6.0%, 4909/81984) than those who received allograft grafts (2.6%, 31330/1201359). The causes of postoperative infection caused by autologous bone grafting are related to the increase of surgical site, the extension of the operation time, and the increase of blood loss [20]. It is suggested that there are two common causes of increased risk of infection after allograft. The first was an unknown infection of the donor, and contamination during acquisition or processing; the second was delayed vascular penetration, slow bone formation, and high bone resorption rate after surgery [21]. Contrary to previous studies, the infection rate of autografts was higher than that of allografts (OR = 6.380 vs OR = 3.215). Although autograft was considered the gold standard for bone grafting, there were some disadvantages, such as the need to increase the surgical site, and duration of surgery and intraoperative blood loss, which might increase the risk of infection [22].

Elevated WBC (white blood cells) counts in trauma patients had been well studied in previous studies. Several studies had explored the prognostic value of white blood cell counts at admission and continuous white blood cell counts during hospitalization in predicting infection during orthopedic surgery [23–25]. In previous studies, postoperative white blood cell count was more likely to be used to predict postoperative infection [26, 27]. We concluded that preoperative WBC counts were an independent risk factor for postoperative infection. It is well known that the increase of WBC count in trauma patients before surgery is caused by the physiological stress of the body’s immune system [28–30]. We speculated that the increase in postoperative infection might be related to immune system disorders following trauma. The innate immune system is not only activated by pathogen-associated molecular patterns but also by trauma, such as fractures or multiple injuries [31, 32]. In this case, so-called risk-associated molecular patterns have been identified as danger signals that mediate early post-traumatic inflammatory responses [33], leading to infectious complications. Therefore, we need to pay enough attention to these patients with an elevated preoperative WBC count, observe the wound regularly and carefully after the operation.

Fractures usually involve soft tissue damage, and consequently, local tissue hypoxia and acidosis in tissue environment will be caused [34]; serum skeletal muscle enzymes are markers of the functional state of muscle tissue, and aspartate aminotransferase (AST) is one of the most useful serum markers of muscle injury [35]. Injury of muscle tissue after fracture can lead to elevated serum AST. In addition, the local tissue environment is in the state of metabolic acidosis after organism injury. With the aggravation of metabolic acidosis, the serum anion gap increases with the increase of serum lactic acid level [36]. Even in some studies, higher serum AG was independently associated with higher levels of inflammatory biomarkers in healthy samples from the general population [37]. In our study, high preoperative serum AST and AG were both independent risk factors for SSI after ORIF of tibial plateau fractures. Therefore, we should pay enough attention to soft tissue injuries and allowed sufficient soft tissue repair via delaying the definite operation by use of temporary external fixation or minimally invasive surgery, to reduce or even avoid SSI.

Although the design of this study was prospective, several limitations had to be mentioned. First, there were several orthopedic trauma surgeons involved in the treatment of tibial plateau fractures, each with their own judgment on the operation. Second, some specific variables, such as smoking status and alcohol consumption, could not be accurately quantified. In addition, some patients might be unwilling to tell us about these bad habits and choose to withhold information. Medical comorbidity information mainly relied on patients’ self-report, and some patients might lack a clear knowledge. Third, it should be pointed out that our hospital was a first-level trauma center and patients might be more severely injured; therefore, the SSI identified in this study could be less representative.

In summary, we observed 25 cases (2.3 %) of SSI in 1108 adult patients with close tibial plateau fractures. Fracture type (Schartzker V–VI), bone grafting (autograft, allograft), white blood cell (>10*109/L), aspartate aminotransferase (>40 U/L), and anion gap (>16 mmol/L) were identified as independent risk factors for SSI. We should pay enough attention to patients who carried one or more of these preoperative factors and adopt more reasonable treatment strategies to avoid the occurrence of SSI.

## Data Availability

The data and materials contributing to this article may be made available upon request by sending an e-mail to the first author.

## Abbreviations

SSI: Surgical site infection
ORIF: Open reduction and internal fixation
SSIOS: Surgical site infection in orthopaedic surgery
